# Alternating Hemiplegia of Childhood: Retrospective Genetic Study and Genotype-Phenotype Correlations in 187 Subjects from the US AHCF Registry

**DOI:** 10.1371/journal.pone.0127045

**Published:** 2015-05-21

**Authors:** Louis Viollet, Gustavo Glusman, Kelley J. Murphy, Tara M. Newcomb, Sandra P. Reyna, Matthew Sweney, Benjamin Nelson, Frederick Andermann, Eva Andermann, Gyula Acsadi, Richard L. Barbano, Candida Brown, Mary E. Brunkow, Harry T. Chugani, Sarah R. Cheyette, Abigail Collins, Suzanne D. DeBrosse, David Galas, Jennifer Friedman, Lee Hood, Chad Huff, Lynn B. Jorde, Mary D. King, Bernie LaSalle, Richard J. Leventer, Aga J. Lewelt, Mylynda B. Massart, Mario R. Mérida, Louis J. Ptáček, Jared C. Roach, Robert S. Rust, Francis Renault, Terry D. Sanger, Marcio A. Sotero de Menezes, Rachel Tennyson, Peter Uldall, Yue Zhang, Mary Zupanc, Winnie Xin, Kenneth Silver, Kathryn J. Swoboda

**Affiliations:** 1 Pediatric Motor Disorders Research Program, Departments of Neurology and Pediatrics, University of Utah, Salt Lake City, Utah, United States of America; 2 Institute for Systems Biology, Seattle, Washington, United States of America; 3 Neurogenetics Unit, Montreal Neurologic Institute and Hospital, McGill University, Montreal Quebec, Canada; 4 Departments of Pediatrics and Neurology, Connecticut Children's Medical Center and University of Connecticut School of Medicine, Hartford, CT, United States of America; 5 Department of Neurology, University of Rochester School of Medicine, Rochester, New York, United States of America; 6 Diablo Valley Child Neurology, an affiliate of Stanford Health Alliance, Pleasant Hill, California, United States of America; 7 Division of Pediatric Neurology, Children's Hospital of Michigan, Wayne State University, Detroit, Michigan, United States of America; 8 Department of Child Neurology, Palo Alto Medical Foundation Redwood City Clinic, Redwood City, California, United States of America; 9 Department of Pediatric Neurology, Children’s Hospital Colorado, University of Colorado Hospital, Aurora, Colorado, United States of America; 10 Departments of Genetics and Genome Sciences, Pediatrics, and Neurology, Case Western Reserve University and University Hospitals Case Medical Center, Cleveland, Ohio, United States of America; 11 Pacific Northwest Diabetes Research Institute, Seattle, Washington, United States of America; 12 Departments of Neuroscience and Pediatrics, University of California San Diego, San Diego, California, United States of America; 13 Department of Epidemiology, The University of Texas M.D. Anderson Cancer Center, Houston, Texas, United States of America; 14 Department of Human Genetics, University of Utah, Salt Lake City, Utah, United States of America; 15 Departments of Pediatrics and Neurology, University College Dublin School of Medicine and Medical Science, Dublin, Ireland; 16 Department of Biomedical Informatics, University of Utah School of Medicine, Salt Lake City, Utah, United States of America; 17 Children’s Neuroscience Centre, Murdoch Childrens Research Institute, University of Melbourne Department of Paediatrics, The Royal Children’s Hospital Melbourne, Parkville Victoria, Australia; 18 Department of Pediatrics, College of Medicine Jacksonville, University of Florida, Jacksonville, Florida, United States of America; 19 Department of Family Medicine, University of Pittsburgh School of Medicine, Pittsburgh, Pennsylvania, United States of America; 20 Stevens Henager College, Salt Lake City, Utah, United States of America; 21 Department of Neurology, University of California San Francisco, San Francisco, California, United States of America; 22 Center for Medical Ethics and Humanities in Medicine, University Of Virginia UVA health system, Charlottesville, Virginia, United States of America; 23 Departement de Neurophysiologie. Hopital Armand Trousseau APHP, Paris, France; 24 Department of Biomedical Engineering, University of Southern California, Los Angeles, California, United States of America; 25 Swedish Neuroscience Institute, Swedish Medical Center, Seattle, Washington, United States of America; 26 Department of Paediatrics and Adolescent Medicine, Juliane Marie Centre, Rigshospitalet, University of Copenhagen, Copenhagen, Denmark; 27 Study Design and Biostatistics Center, University of Utah, Salt Lake City, Utah, United States of America; 28 Department of Neurology, Children’s Hospital Orange County, and Department of Pediatrics, University of California, Orange, California, United States of America; 29 Center for Human Genetic Research, Department of Neurology, Massachusetts General Hospital, Boston, Massachusetts, United States of America; 30 Departments of Pediatrics and Neurology, University of Chicago and Comer Children's Hospital, Chicago, Illinois, United States of America; Shenzhen Institutes of Advanced Technology, CHINA

## Abstract

Mutations in *ATP1A3* cause Alternating Hemiplegia of Childhood (AHC) by disrupting function of the neuronal Na+/K+ ATPase. Published studies to date indicate 2 recurrent mutations, D801N and E815K, and a more severe phenotype in the E815K cohort. We performed mutation analysis and retrospective genotype-phenotype correlations in all eligible patients with AHC enrolled in the US AHC Foundation registry from 1997-2012. Clinical data were abstracted from standardized caregivers’ questionnaires and medical records and confirmed by expert clinicians. We identified *ATP1A3* mutations by Sanger and whole genome sequencing, and compared phenotypes within and between 4 groups of subjects, those with D801N, E815K, other *ATP1A3* or no *ATP1A3* mutations. We identified heterozygous *ATP1A3* mutations in 154 of 187 (82%) AHC patients. Of 34 unique mutations, 31 (91%) are missense, and 16 (47%) had not been previously reported. Concordant with prior studies, more than 2/3 of all mutations are clustered in exons 17 and 18. Of 143 simplex occurrences, 58 had D801N (40%), 38 had E815K (26%) and 11 had G937R (8%) mutations. Patients with an E815K mutation demonstrate an earlier age of onset, more severe motor impairment and a higher prevalence of status epilepticus. This study further expands the number and spectrum of *ATP1A3* mutations associated with AHC and confirms a more deleterious effect of the E815K mutation on selected neurologic outcomes. However, the complexity of the disorder and the extensive phenotypic variability among subgroups merits caution and emphasizes the need for further studies.

## Introduction

Alternating Hemiplegia of Childhood (AHC, MIM#614820) is a rare and complex neurodevelopmental disorder described initially by Verret and Steele in 1971, and named for the characteristic recurrent attacks of hemiplegia that affected first one side of the body, then another. However, episodes of paroxysmal neurologic dysfunction in this unusual disorder encompass a wide range of abnormal movements and episode types ranging from hemiplegia to quadriplegia to dystonia, and lasting from minutes to hours or even days. A variety of external or emotional stressors trigger the onset or worsening of paroxysmal symptoms, but they are consistently relieved by sleep, whether pharmacologically or naturally induced [1,2]. Although rare familial cases with autosomal dominant inheritance have been reported [3–6], AHC is predominantly a sporadic disorder. Estimated incidence of the classic form of the disease is 1 in one million: males and females are affected in roughly equal numbers. The variability and the complexity of presenting symptoms have historically led to considerable delays in diagnosis, which was until recently based solely on carefully conceived clinical diagnostic criteria. These criteria specified an infantile onset (< 18 months) of recurrent paroxysmal episodes of hemiplegia, dystonia and ocular movement abnormalities, and were ultimately characterized by the appearance of additional fixed neurologic signs and symptoms including chorea, dysarthria, dyskinesia, ocular apraxia, ataxia and global developmental delay [2,7,8]. The variable course of the disease and limited longitudinal follow-up and neuropathologic data have led to ongoing speculation as to whether or not AHC represents a static neurodevelopmental or a progressive disorder [9–11].

The pathophysiology of AHC was totally unknown until the recent identification of mutations in *ATP1A3*, which encodes a neuron specific sodium/potassium ATPase involved in the regulation of neuronal excitability [12]. We and others initially identified de novo recurrent mutations in *ATP1A3* by performing whole exome studies in trios in a series of simplex AHC patients. These initial studies indicated that more than 2/3 of patients had de novo mutations, confirming a causative role of ATP1A3 in the pathogenesis of AHC, and that two mutations accounted for most of the confirmed cases [13–21]. *ATP1A3* was previously shown to be mutated in Rapid-Onset Dystonia-Parkinsonism (RDP, DYT12, MIM#128235), a rare neurological disorder characterized by abrupt onset of dystonia and triggered by emotional or physical stress [22–37]. More recently, mutations were identified in another childhood onset condition called CAPOS syndrome(MIM#601338) with paroxysmal neurological symptoms overlapping with AHC and DYT12 but with distinctive features including pes cavus, optic atrophy and sensorineural hearing loss [38,39]. To date, mutations in *ATP1A3* for these different phenotypes have proved largely non-overlapping, indicating that genotypes may be predictive of phenotype in these allelic disorders, at least for patients presenting with the classic phenotypes as defined using strict clinical diagnostic criteria. Since *ATP1A3* mutations were reported in patients with AHC in 2012, the increasing numbers of patients with overlapping or unique phenotypes have led to use of the broader term “*ATP1A3*-related neurologic disorders.” However, it is notable that, to date, patients with the classically described phenotypes associated with AHC and RDP share very little overlap in the precise mutations identified. This underscores the importance of genotype-phenotype studies and that will undoubtedly provide clues to understand the unique pathogenesis of these clinical syndromes.

Here we present the results of a genetic and clinical study of the largest AHC cohort reported to date. This cohort includes 187 AHC patients enrolled since 1997 in the US AHC foundation (AHCF) registry and biobank at the University of Utah. We identified 34 unique mutations in *ATP1A3* among 154 affected individuals, accounting for 82% of cases (154/187) and confirming the previously observed high frequency of the D801N and E815K mutations among AHC patients. In a subset of patients with complete clinical data, we performed a retrospective genotype-phenotype correlation study to evaluate the influence of the two most frequently observed mutations on selected outcomes in patients with a clinical diagnosis of AHC.

## Patients

One hundred eighty seven patients (82 males and 105 females) with AHC enrolled in the registry from 1997 to 2012 and provided DNA samples and clinical information for this analysis. Subjects were predominantly from the USA (108) but also from Australia (6), Brazil (2), Canada (7), Chile (1), Croatia (1), Czech Republic (2), Denmark (2), France (27), Iceland (1), Ireland (2), India (2), Israel (1), Italy (5), Kenya (1), Mexico (1), the Netherlands (1), New Zealand (1), Puerto Rico (4), Spain (4), Sweden (2), Turkey (1), and the United Kingdom (4). Clinical diagnosis of AHC had been confirmed in all cases by a neurologist based on published diagnostic criteria for AHC; a second review of available history and records was performed by authors KJS, KS or MS. Typical cases, defined as “clinically definite,” fulfilled the diagnostic criteria as originally defined by Bourgeois et al.^2^ Atypical cases deemed “clinically probable” by the referring physician were also included in this analysis and included subjects with onset of first symptoms > 18 months of age but whose phenotype fulfilled all other diagnostic criteria for AHC.

One hundred sixty-nine patients were simplex cases, and DNAs of both parents were available for 70 (41%). The cohort included two sets of monozygotic twins who were concordantly affected. Fourteen subjects in 5 multiplex pedigrees, 2 with dominant inheritance, were also included in the present analysis. Patients and their parents gave informed written consent for clinical data and DNA storage and analysis. Ethics approval was obtained from the Institutional Review Board at the University of Utah. Among the 187 patients, 13 have been included in the genetic study published by Heinzen et al in 2012 [12].

## Methods

DNA was extracted from peripheral venous blood using QIAmp DNA minikit (Qiagen, Hilden, Germany). *ATP1A3* sequencing was performed on DNA from AHC probands and family members using the Sanger method after PCR amplification of the 23 coding exons and their flanking regions with intronic primers as defined by Heinzen et al (reference sequence ENSG00000105409/NCBI:NM_001256214.1) [12]. PCR products were purified with ExoSAP-IT (Affymetrix, Santa Clara, CA) and sequenced using the BigDye3.1 terminator method on an ABI PRISM 3730xl Genetic Analyzer (Applied Biosystems, Foster City, CA). Sequences were analyzed with Sequencher 4.9 Software (Genes Codes Corporation, Ann Harbor, MI) and DNA variants were described using ALAMUT Software (Interactive-Biosoftware, France). Evaluation of pathogenicity of the DNA variants was made using the Polymorphism Phenotyping (Polyphen) program (http://genetics.bwh.harvard.edu/pph/), the Provean program (http://provean.jcvi.org/index.php) and the Human Splicing Finder tool (http://www.umd.be/HSF/). Detection of the proband's mutation in the DNA of the parents was performed by Sanger sequencing of the mutated exon. DNA from an additional 30 probands was examined via whole genome sequencing on the CGI platform (Complete Genomics Inc., Mountain View, CA), including 25 parent-child trios and an additional 5 multiplex AHC pedigrees. Sequences were aligned to the GRCh37 (hg19) human reference genome. Variants were evaluated using QIAGEN’s Ingenuity Variant Analysis software. We tested for presence and frequency of identified variants in the Kaviar database (version 141029), which integrates data from 1000 genomes, the Personal Genome Project, ESP6500, dbSNP and several other public resources, and thousands of private genome sequences [40].

For the clinical phenotype portion of the study, information was collected using standardized questionnaires completed by the parent or caregiver first, and then validated by the physician. For some patients, follow-up information was available from medical records, subsequent in-person evaluations at one of several national family meetings sponsored by the AHCF, or via follow-up telephone interviews. Main characteristics of the subset of AHC patients with complete clinical data are summarized in Table 1. Clinical characteristics were compared among a cohort of 164 subjects with and without *ATP1A3* mutations. We also compared clinical data between individual patient cohorts with the two most frequent mutations, D801N and E815K, and to those with other *ATP1A3* mutations.

**Table 1:** Summary of 164 AHC patients included in the genotype-phenotype correlation study.

*ATP1A3* mutations	E815K	D801N	Other Mutations	No Mutation
**Total Number of Subjects**	31	58	49	26
**Females**	16 (51.6%)	36 (62.1%)	25 (51.0%)	15 (57.7%)
**Males**	15 (48.4%)	22 (37.9%)	24 (49.0%)	11 (42.3%)
**Mean Age at Onset, in months (standard deviation)**	1.9 (3.3)	4.2 (4.0)	5.4 (7.9)	12.2 (12.5)
**Mean Age at Last Evaluation, in years (standard deviation)**	7.5 (5.2)	12.0 (7.7)	13.4 (10.1)	9.5 (5.9)

## Statistical Analysis

Descriptive analyses were used to examine the characteristics of the study population by mutation subtype. Since the age at onset was not normally distributed, quantile regression, more specifically median regression with gender adjustment, was used to estimate the differences in age at onset between E815K mutation, the D801N mutation and other mutations. Non-parametric survival analysis and log-rank tests were conducted to estimate and compare the curves of the cumulative probabilities of acquiring the gross motor skills of unsupported sitting and independent walking between the same mutation group pairs. Logistic regression was used to quantify how strongly the presence of an E815K mutation was associated with the occurrence of epilepsy. All the analyses were performed using the SAS version 9.2 statistical package (SAS Institute, Cary, NC). Statistical significance was assessed assuming a 0.05 significance level and a two-sided alternative hypothesis.

## Results

### Genetic analysis

**Fig 1:**
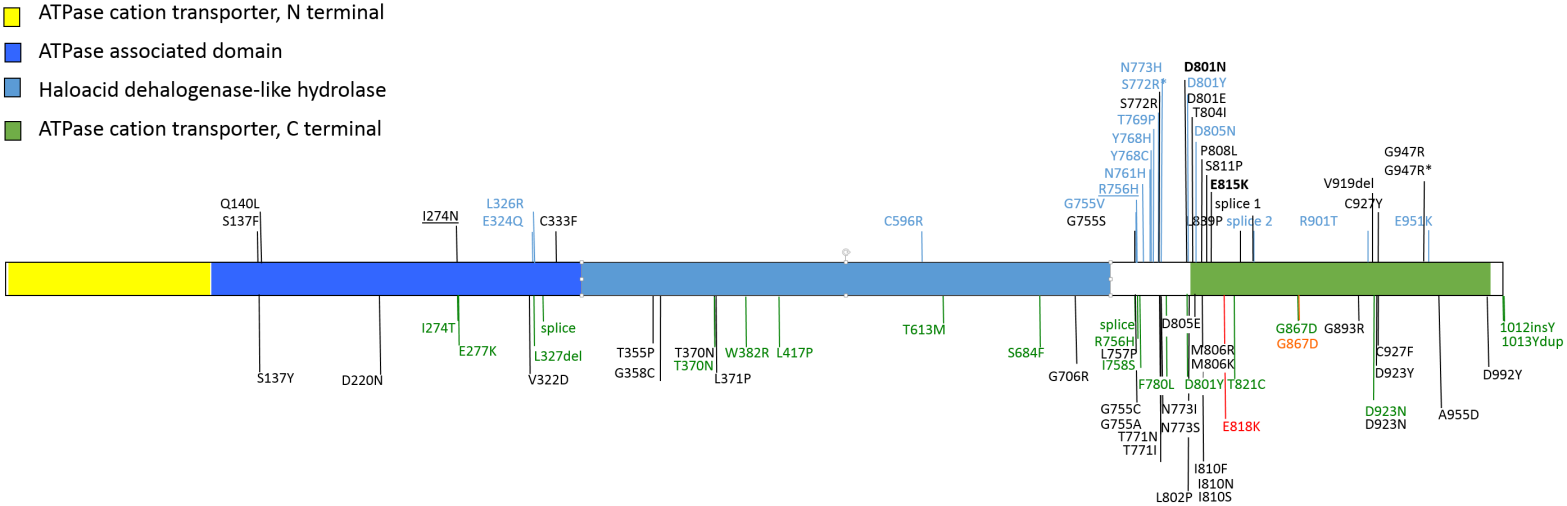
Schematic representation of *ATP1A3* mutations. Mutations identified in our cohort are indicated above the gene; all the mutations previously published are indicated in black; novel mutations are indicated in light blue; mutations identified in multiplex cases are underlined; mutations reported in DYT12 are indicated in green; the mutation reported in CAPOS syndrome is indicated in red. The mutation associated with a phenotype combining features of both AHC and RDP is in orange. The 2 most common mutations are in bold. Asterisks mean that 2 different nucleotide changes have been identified for these protein variants.

We identified 34 distinct *ATP1A3* mutations in 154 patients, including 68 males and 86 females. One hundred forty three were simplex cases, 4 patients were two sets of monozygotic twins, and 7 patients were members of 2 families with dominant inheritance (Table 2). The mutations were all heterozygous and predominantly missense (31/34), located in the coding sequence of 9 exons of *ATP1A3*, with hot spots in exons 17 and 18 suggested by 3 recurrent common mutations. A deletion of 3 nucleotides in exon 20, predicting deletion of a single amino acid from the ATP1A3 protein sequence, was found in 2 patients. Two intronic mutations at the donor site of exon 18 were found in 3 patients, predicting abnormal splicing of this intron (Fig 1). All the missense mutations identified in this cohort were predicted to be damaging (see S1 Table). We tested for their presence and frequency in the Kaviar database and concluded that these variants have never been observed other than in AHC patients. No mutation was detected in the exonic sequence of *ATP1A3* in 33 AHC patients.

**Table 2:** Summary of the 187 patients included in the genetic study.

Number of patients	Simplex cases	Homozygous twins	Multiplex cases	Total
**With ATP1A3 mutations**	143	2 twins, 2 twins	4 of 1 family, 3 of 1 family	154
**Without ATP1A3 mutation**	26	0	3 of 1 family, 2 of 1 family, 2 of 1 family	33
**Total**	169	4	14	187

The predominant mutation, D801N, was found in 40% of the simplex patients (58/143) and the mutation E815K in 26% (38/145); neither of these mutations were observed in multiplex cases (see S1 Table). In the 70 simplex cases for which DNA was available from both parents, all were confirmed to be de novo mutations. One dominant family with 4 affected family members was previously reported [3]. In another family, a heterozygous mutation (R756H) segregates with the disease phenotype in an affected mother and her two affected children. No *ATP1A3* mutation was found in 3 other multiplex families. Of the 34 mutations identified in this cohort, 16 are novel and 18 were previously reported in AHC patients [13–21].

### Phenotype analysis and correlations

Data were extracted from standardized medical questionnaires that had been previously entered into a RedCAP database associated with the registry. Data for the current analysis were selected according to the reliability of documentation, their availability in most patients, and their perceived importance as indicated by emphasis in other recent publications. The variables include: 1) the age of the first paroxysmal symptom or sign (i.e., ocular movements, dyskinesia, or unilateral or bilateral paralysis or seizure-like episode); 2) the age at which the child achieved unsupported sitting; 3) the age at which the child achieved independent walking, and 4) a history of status epilepticus and/or sudden unexplained death during the time of follow up.

**Fig 2:**
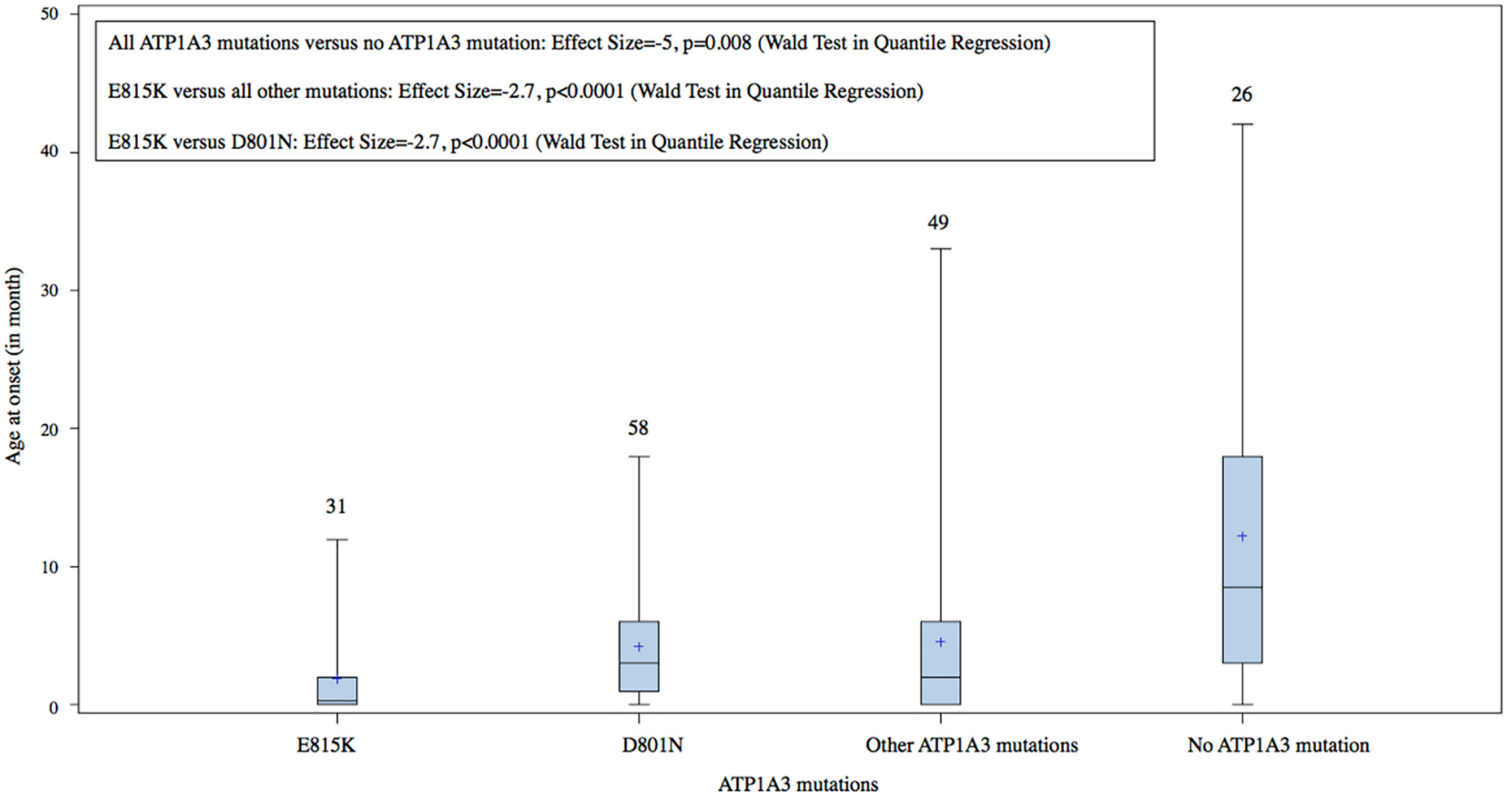
Ages at onset of AHC in each group of patients defined by their genotype. The horizontal lines in the boxes indicate the 25th percentile (bottom), the median (middle) and the 75 percentile (top) values. Crosses indicate the mean values. Numbers of patients analyzed in each group are indicated above the boxes.

**Fig 3:**
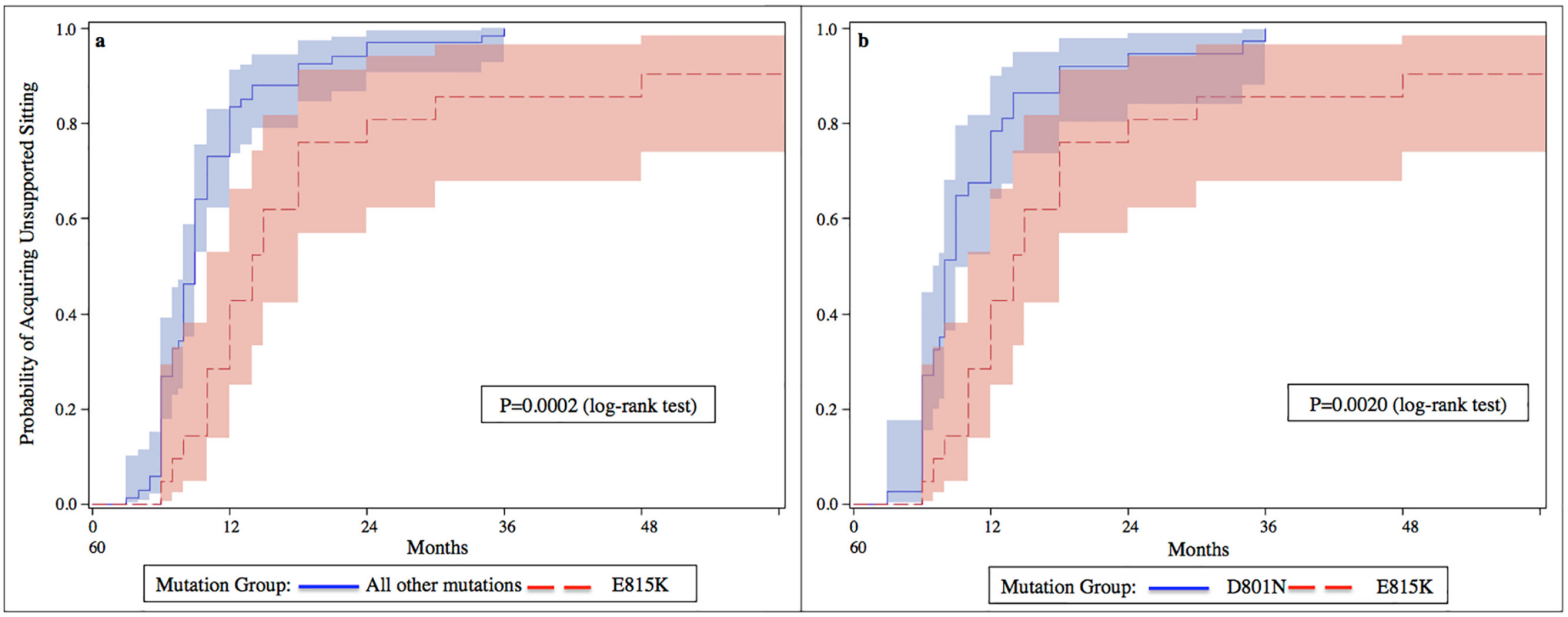
Ages at unsupported sitting acquisition in each group of patients defined by their genotype. Cumulative probability of acquiring unsupported sitting by patients presenting the E815K mutation, compared to patientsmutation (3b). Patients with the E815K mutation are likely to gain unsupported sitting at a later age than patients in each of the other groups (respectively P = 0.0002 and P = 0.0020).

**Fig 4:**
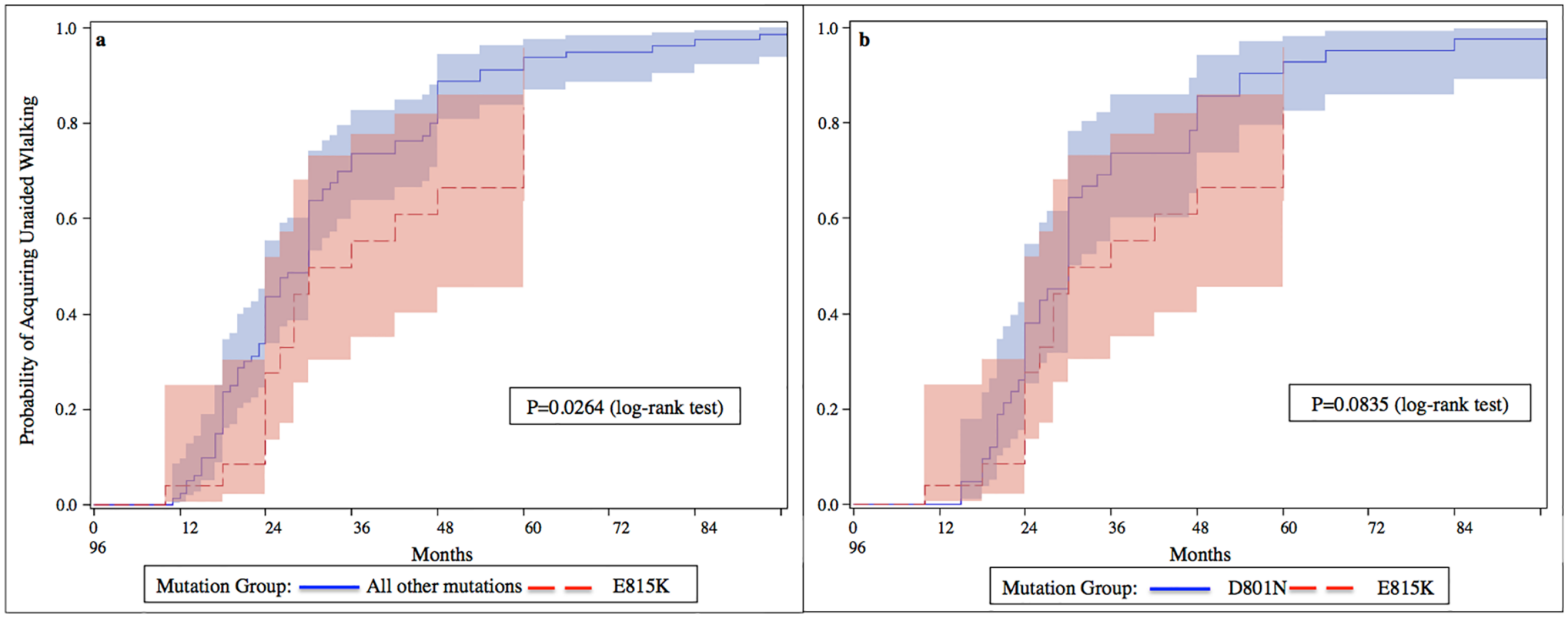
Ages at unaided walking acquisition in each group of patients defined by their genotype. Cumulative probability of acquiring unaided walking by patients presenting the E815K mutation, compared to patients with all other *ATP1A3* mutations (4a) and to patients with the D801N mutation (4b). Patients with the E815K mutation are likely to gain unaided walking at a later age than patients in each of the other groups (respectively P = 0.0264 and P = 0.0835).

All patients with the D801N or E815K mutation had onset of their first paroxysmal symptoms by 18 months of age. The 6 patients presenting an atypical late onset of the disease had a rare *ATP1A3* variant or did not have any detectable *ATP1A3* mutation. Those patients with a mutation in *ATP1A3* had a significantly earlier median age at onset (5 months earlier) than for those without a mutation (P-value = 0.008). When contrasting the most common mutations, the age at onset for patients with the E815K mutation was 2.7 months earlier than those with other types of *ATP1A3* mutations (P-value<0.0001), as well as 2.7 months earlier than those with the D801N mutation (P-value<0.0001) (Fig 2). Patients with E815K mutations achieved unsupported sitting later than those with the D801N mutation (P-value = 0.0020) and later than those with other mutations (P-value = 0.0002) (Fig 3). Patients with E815K mutations were also more likely to achieve independent walking later than those with other mutations (P-value = 0.0264) (Fig 4). Finally, patients with E815K mutation were almost 3 times more likely to present with status epilepticus during the course of the disease than the patients with other types of *ATP1A3* mutations (P-value = 0.0206. Table 3)

**Table 3:** Odds ratio for occurrence of status epilepticus in AHC patients with different *ATP1A3* mutations.

ATP1A3 mutations	Odds Ratio	Confidence Interval	P-value
**E815K vs. all other mutations**	2.73	(1.166, 6.370)	0.021
**E815K vs. D801N**	1.12	(1.193, 7.919)	0.020
**D801N vs. all other mutations**	0.54	(0.252,1.141)	0.106

## Discussion

Since mutations in *ATP1A3* were first identified in 2012 in patients with AHC, the prevalence of mutations in sporadic patients meeting classic diagnostic criteria for AHC has ranged from 70% to 100% across a variety of ethnic backgrounds [12–20]. The 2 most common mutations, D801N and E815K, account for more than 60% of all ATP1A3 mutations resulting in an AHC phenotype [12]. We extend these observations in this large cohort, confirming a similar prevalence among our AHC cases for the 2 most common mutations, and confirming that most mutations causing AHC seem to occur in a region located between the haloacid-dehalogenase-like-hydrolase and the C-terminal-ATPase domains, close to the transmembrane domains of the protein. The 16 novel mutations we report here, however, effectively double the number of identified mutations associated with an AHC phenotype, and further extend the full range of identified mutations.

Although the majority of AHC mutations identified here did not overlap with mutations previously reported in association with an RDP phenotype, we found two mutations (D801Y and R756H) previously reported with RDP phenotypes, although the latter familial occurrence has unique features, with some that overlap with both disorders. Clinical features of these patients are summarized in S2 Table. Such variable expressivity has been recently reported for two other mutations (T370N and D923N) in *ATP1A3*-related disorders [19, 21]. Rosewich et al. noted that specific *ATP1A3* mutations were associated with certain features characteristic of both AHC and RDP phenotypes [19,41].

Despite a high rate of mutation detection in *ATP1A3* in our study (82%), we did not identify mutations in all of the patients. Genome sequencing of the *ATP1A3* negative cohort also failed to identify obvious variants in genes associated with familial hemiplegic migraine (ATP1A2, SLC1A3/EEAAT1, CACNA1A), in agreement with prior investigations [18,42–45].

Previous studies showed that patients with early onset of AHC symptoms tended to have a more severe clinical course [7,11]. Our genotype-phenotype correlation study shows an earlier age at disease onset and a significantly higher incidence of status epilepticus in the E815K mutation group. Ishii et al. reported similar observations in Japanese patients with E815K mutations [14].

The significant delay in achievement of gross motor milestones observed here in patients with the most common *ATP1A3* mutations is also concordant with previous findings. However, patients within cohorts with the same mutation (for example, the 58 D801N-mutation patients and the 31 E815K-mutation patients with complete clinical data) presented a wide range of age of onset (0–12 months and 0–16 months, respectively), developmental delay, and overall neurologic morbidity. In comparing our E815K cohort to the Japanese E815K cohort, outcomes appear to be overall consistently more severe in the latter, suggesting the possible influence of epigenetic or additional unknown genetic factors that modify expression of the disease. However, we had less complete data for those with E815K than with D801N mutations in our cohort. Finally, we observed an earlier median age at onset in patients with any *ATP1A3* mutation (D801N, E815K or other) relative to patients without such mutations.

During the last 10 years, several functional studies have attempted to decipher the pathophysiology of ATP1A3 related disorders and to explain the important phenotypical variability of these diseases [1, 46–53] Using cellular models, Heinzen et al showed that D801N, E815K and 3 other AHC mutations did not decrease the level of expression of the alpha 3 subunit of the Na+/K+ATPase but significantly decreased the ATPase activity [12]. This functional defect was quantitatively similar for E815K as for the other mutations, suggesting that the ATPase activity defect solely could not explain the higher severity of the E815K associated phenotype. The other major function of the alpha subunit of the Na+/K+ ATPase is the ion transport across the cell membrane, in a process called forward cycling: three Na+ go out of the cell while two K+ go in, using ATP hydrolysis, with a concomitant passive inward transport of protons [54]. Interestingly, the majority of the mutations associated with AHC are located in the transmembrane domains of the Na^+^/K^+^ ATPase alpha subunit, suggesting a specific impact of these mutations on ion binding and transport. Using electrophysiological techniques on Xenopus Levis oocytes, Li et al investigated the consequences of D801N, E815K and G947R mutations on Na+, K+ and H+ flows [55]. They showed that these 3 mutants were associated with a loss of ion transport with a strong dominant negative effect, suggesting that the loss of forward cycling is a pathological mechanism in AHC. The level of impact on Na^+^ and K^+^ transport was similar for D801N, E815K and G947R but the proton transport was significantly more profoundly reduced by the E815K than by the two other mutations. The severity of the phenotype associated with the E815K mutation is possibly the consequence of a more profound intracellular alkalosis. Intracellular proton concentration is known to be an important modulator of neuronal excitability and such a change may certainly have severe consequences on neuronal function.

Prediction of the course and severity of the disease using genotype information is a major aim of genotype-phenotype correlation studies. This information is essential for the evaluation of new therapeutics, to provide a more accurate prognosis to patients and caregivers, and to help develop the most appropriate guidelines for care management. Currently, however, the reliability and consistency of tools used to evaluate the severity of AHC are problematic. Panagiotakaki et al. designed specific indexes of paroxysmal and non-paroxysmal disability for AHC evaluation and used these tools in a 2-year retrospective and prospective study of 157 patients [10]. Still, this remarkable study did not demonstrate evidence of disease progression, in contrast to observations from the Japanese patient cohort [15]. Phenotype-genotype correlation from our analysis and others suggests that patients with E815K mutations are at greater risk of an acute clinical decompensation. Thus, even as our understanding of the genetics and pathophysiology of AHC increases, there is a continued need for comprehensive longitudinal natural history studies of AHC.

## Conclusion

Further progress in our understanding of the pathophysiology of AHC and the broader spectrum of *ATP1A3* related phenotypes will require that a number of initiatives proceed in parallel, including 1) close collaboration among patients, physicians and researchers to facilitate early diagnosis via molecular studies; 2) early enrollment of the majority of patients in registries and prospective longitudinal studies; 3) a strong international collaboration to ensure uniform collection of data to better understand disease pathogenesis and outcome; and 4) the identification and implementation of valid, reliable, and sensitive outcome measures for future use in clinical trials to assess promising therapies. Such outcomes measures should include (a) caregiver questionnaires and patient or caregiver reported outcomes, (b) standardized patient assessments of motor and cognitive function over a wide range of age and disease severity, and (c) tools for tracking AHC episode characteristics such as duration, frequency, and severity. Achieving such aims will enable progress towards an improved understanding of this complex and devastating disease, and help to ensure access to clinical trials and ultimately, truly effective therapies.

## Supporting Information

S1 Table Genetic study summary table. Heterozygous *ATP1A3* mutations and protein modifications found in AHC patients in the AHCF registry enrolled from 1997 to 2012.
(PDF)

S2 Table D801Y and R756H associated phenotypes. Summary of the clinical informations from the patients presenting the D801Y and the R756H mutations.
(PDF)
