# Correction: Alternating Hemiplegia of Childhood: Retrospective Genetic Study and Genotype-Phenotype Correlations in 187 Subjects from the US AHCF Registry

**DOI:** 10.1371/journal.pone.0137370

**Published:** 2015-08-31

**Authors:** Louis Viollet, Gustavo Glusman, Kelley J. Murphy, Tara M. Newcomb, Sandra P. Reyna, Matthew Sweney, Benjamin Nelson, Frederick Andermann, Eva Andermann, Gyula Acsadi, Richard L. Barbano, Candida Brown, Mary E. Brunkow, Harry T. Chugani, Sarah R. Cheyette, Abigail Collins, Suzanne D. DeBrosse, David Galas, Jennifer Friedman, Lee Hood, Chad Huff, Lynn B. Jorde, Mary D. King, Bernie LaSalle, Richard J. Leventer, Aga J. Lewelt, Mylynda B. Massart, Mario R. Mérida, Louis J. Ptáček, Jared C. Roach, Robert S. Rust, Francis Renault, Terry D. Sanger, Marcio A. Sotero de Menezes, Rachel Tennyson, Peter Uldall, Yue Zhang, Mary Zupanc, Winnie Xin, Kenneth Silver, Kathryn J. Swoboda

There is an error in the name of the G947R mutation in the 8^th^ sentence of the abstract. The correct sentence is: “Concordant with prior studies, more than 2/3 of all mutations are clustered in exons 17 and 18. Of 143 simplex occurrences, 58 had D801N (40%), 38 had E815K (26%) and 11 had G947R (8%) mutations.“

There is an error in the nucleotide in the 17^th^ row in S1 Table. The authors have provided a corrected version of S1 Table below.

## Supporting Information

S1 Table Genetic study summary table. Heterozygous *ATP1A3* mutations and protein modifications found in AHC patients in the AHCF registry enrolled from 1997 to 2012.
(PDF)
