# Supplementary material for: Alternating Hemiplegia of Childhood: Retrospective Genetic Study and Genotype-Phenotype Correlations in 187 Subjects from the US AHCF Registry
Source: PLoS One. 2015 May 21;10(5):e0127045. doi: 10.1371/journal.pone.0127045 (PMC4440742; doi:10.1371/journal.pone.0127045)
Supplement: S1 Table — Heterozygous ATP1A3 mutations and protein modifications found in AHC patients in the AHCF registry enrolled from 1997 to 2012. (PDF) [file pone.0127045.s001.pdf]

S1_Table: Genetic study summary table. Heterozygous *ATP1A3* mutations and protein modifications found in AHC patients in the AHCF registry enrolled from 1997 to 2012**.**

Nucleotide Δ	Protein Δ	Position on Chromosome 19	Exon	Patient Number	Predicted Consequence
**410 C>T**	S137F	g.42,490,329	5	1	probably damaging, 1.00
**419A>T**	Q140L	g.42,490,320	5	1	probably damaging, 0.99
**821T>A**	I274N		8	4#	probably damaging, 1.00
**972G>C**	**Q324D**	g.42,489,093	8	1	probably damaging, 0.99
**977T>G**	**L326R**	g.42,489,086	8	1	probably damaging, 0.99
**998G>T**	C333F	g.42, 486, 254	9	1	probably damaging, 1.00
**1786T>C**	**C596R**	g.42,482,323	13	1	probably damaging, 1.00
**2263G>A**	G755S	g.42,479,781	16	5	probably damaging, 0.99
**2264G>T**	**G755V**	g.42,474,694	17	1	probably

					damaging, 1.00
**2267G>A**	**R756H***	g.42,474,691	17	3#	probably damaging, 1.00
**2281A>C**	**N761H**	g.42,474,677	17	1	probably damaging, 0.99
**2302T>C**	**Y768H**	g.42,474,656	17	1	probably damaging, 0.98
**2303A>G**	**Y768C**	g.42,474,655	17	1	probably damaging, 0.99
**2305A>C**	**T769P**	g.42,474,653	17	1	probably damaging, 0.99
**2314A>C**	**S772R**	g.42,474,644	17	1	possibly damaging, 0.94
**2316C>A**	S772R	g.42,474,642	17	1	possibly damaging, 0.94
**2318A>C**	**N773H**	g.42,474,640	17	1	probably damaging, 0.99
**2401G>A**	D801N	g.42,474,557	17	58+2*	probably damaging, 0.99
**2401G>T**	**D801Y***	g.42,474,557	17	1	probably damaging, 0.99
**2403T>A**	D801E	g.42,474,555	17	2*	possibly damaging, 0.79
**2411C>T**	T804I	g.42,474,547	17	2	probably

					damaging, 0.99
**2413G>A**	**D805N**	g.42,474,545	17	1	probably damaging, 0.99
**2423C>T**	P808L	g.42,474,456	18	1	probably damaging, 0.98
**2431T>C**	S811P	g.42,474,448	18	3	probably damaging, 0.98
**2443G>A**	E815K	g.42,474,436	18	38	probably damaging, 1.00
**2516T>C**	L839P	g.42,474,363	18	1	probably damaging, 0.99
**2542+1G>A**	splice site	g.42,474,336		1	splice site broken, -29.76%
**2542+2T>C**	**splice site**	g.42,474,335		2	splice site broken -29.76%
**2702G>C**	**R901T**	g.42,473,054	20	1	probably damaging, 0.99
**2751_2753 delTGT**	V919del	g.42,473,005-42,473,003	20	2	deleterious, -8.77%
**2780G>A**	C927Y	g.42,472,976	20	1	probably damaging, 0.98
**2839G>A**	G947R	g.42,471,896	21	10	probably damaging, 0.99

**2839G>C**	G947R	g.42,471,896	21	1	probably damaging, 0.99
**2851G>A**	**E951K**	g.42,471, 884	21	1	probably damaging, 0.99

Scoring of predicted consequence was obtained from PolyPhen for missense, Human Splicing Finder for intronic mutations and Provean for deletions. AHC=Alternating Hemiplegia of Childhood; **new, previously unreported mutations in bold**; #multiplex cases; *monozygotic twin pair, **previously reported but with RDP phenotype
