# Supplementary material for: Alternating Hemiplegia of Childhood: Retrospective Genetic Study and Genotype-Phenotype Correlations in 187 Subjects from the US AHCF Registry
Source: PLoS One. 2015 May 21;10(5):e0127045. doi: 10.1371/journal.pone.0127045 (PMC4440742; doi:10.1371/journal.pone.0127045)
Supplement: S2 Table — Summary of the clinical informations from the patients presenting the D801Y and the R756H mutations. (PDF) [file pone.0127045.s002.pdf]

S2_Table: D801Y and R756H associated phenotypes. Summary of the clinical informations from the patients presenting the D801Y and the R756H mutations

	**Patient presenting the D801Y mutation**
	Male, 32years old.
**Birth**	Normal
**Development**	Motor milestones and speech acquisition normal<br>At 14 months: episodes of fine tremor of the extremities
**Childhood and Adolescence**	Recurrent episodes of right or left sided hemiplegia, induced by excitement or fear, usually preceded by severe headaches and associated with dystonia,<br>Less frequent episodes of quadriplegia<br>No effect of Flunarizine nor Inderal<br><br>Between the spells: Ataxia, right hemiparesia, right sided athetosis not improved by Sinemet, fluctant dysarthria, abnormal eye movements<br><br>Mental development: IQ=123 at 4y3M and 92 at 12yo
**At 30 yo**	First seizure (Grand Mal)
**Last examination at 31 yo**	Decreased frequency of the hemiplegic/dystonic episodes<br>Dysarthria<br>Tremor between the spells<br>Mild ataxia and uncoordination but walks independently<br><br>Graduated from High School but decreased mental skills

	Patients presenting the R756H mutation	
	Female, 5.5 years old	Female, 11.5 years old
**Birth**	Normal	Normal
**Development**	Motor milestones and speech slightly delayed by 3-4 months	Motor milestones and speech acquisition normal
**Childhood**	Febrile seizure at 2.5 yo with residual persistent right sided hemiplegia and ataxia<br><br>Recurrence of seizure at 3 yo, followed by dysarthria, dysphagia.	Febrile seizure at 2 yo<br><br>First episode of hemiplegia or dystonia at 3.5 yo
**Last examination**	**at 3yo**<br>Right sided hemiplegia<br>Truncal ataxia needing assistance for walking and sitting<br>Poor speech	**at 9.5 yo**<br>Ataxia and dysmetria<br>Dysarthria<br><br>Learning disability
